# AAV‐Tau injection in the substantia nigra as a rapid and robust mouse model of tauopathies with Parkinsonian features

**DOI:** 10.1002/alz70855_102244

**Published:** 2025-12-23

**Authors:** Anastasia Noel, Elodie Brison, Barry J Bedell

**Affiliations:** ^1^ Biospective Inc, Montreal, QC, Canada

## Abstract

**Background:**

Tau pathology in the substantia nigra has been recognized as a key feature in tauopathies with Parkinsonian features, where abnormal tau protein accumulation is associated with neurodegeneration. Understanding the role of tau in this brain region is essential for developing better models and therapies for tauopathies. Adeno‐associated virus (AAV) vectors are able to effectively deliver tau protein into specific brain regions, thereby enabling the study of tau‐related neurodegeneration in vivo. Here, we characterized a novel mouse model utilizing AAV‐induced human wild‐type tau expression specifically targeting the substantia nigra, designed to study tau‐related neurodegeneration in the midbrain, including dopaminergic neurons.

**Method:**

Wild‐type C57BL/6 mice underwent unilateral stereotaxic injection of an AAV vector overexpressing wild‐type human (2N4R) tau (AAV‐Tau) or an empty (control) AAV vector into the substantia nigra pars compacta at 9 (young) or 48 (aged) weeks‐of‐age. Hindlimb clasping and nesting scores were assessed weekly to monitor the progression of gross motor deficits over time. Asymmetric locomotor behavior was assessed at 6 and 10 weeks post‐injection (WPI) of the AAVs via the tail suspension swing test and the cylinder test. The rotarod test was used to evaluate motor coordination and balance at 7 and 11 WPI. Regional neuroanatomical volumes were assessed via automated image analysis of in vivo anatomical MRI scans acquired at 12 WPI using a 7T preclinical MRI scanner.

**Result:**

AAV‐Tau‐injected mice showed early spontaneous progressive motor dysfunction, including locomotor asymmetry, motor coordination and balance impairments, and hindlimb clasping deficits. The AAV‐Tau‐induced phenotype was exacerbated in aged mice compared to young animals, and was associated with increased spontaneous contralateral rotations during the cylinder test. Significantly decreased regional neuroanatomical volumes (e.g. midbrain, striatum) were observed in the ipsilateral hemisphere when compared to the contralateral hemisphere in the AAV‐Tau animals.

**Conclusion:**

The early development of motor deficits and regional brain atrophy observed in AAV‐Tau‐injected mice makes this translational model a valuable and reliable tool for preclinical efficacy studies aimed at accelerating the development of disease‐modifying therapeutic interventions for tauopathies with Parkinsonian features, such as Progressive Supranuclear Palsy (PSP) and Corticobasal Degeneration (CBD).

